# BTB/POZ zinc finger protein ZBTB16 inhibits breast cancer proliferation and metastasis through upregulating *ZBTB28* and antagonizing *BCL6*/*ZBTB27*

**DOI:** 10.1186/s13148-020-00867-9

**Published:** 2020-06-09

**Authors:** Jin He, Mingjun Wu, Lei Xiong, Yijia Gong, Renjie Yu, Weiyan Peng, Lili Li, Li Li, Shaorong Tian, Yan Wang, Qian Tao, Tingxiu Xiang

**Affiliations:** 1 Department of Oncology, The First Affiliated Hospital of Chongqing Medical University, Chongqing, China; 2 Key Laboratory of Molecular Oncology and Epigenetics, The First Affiliated Hospital of Chongqing Medical University, Chongqing, China; 3 Institute of Life Science, Chongqing Medical University, Chongqing, China; 4 Cancer Epigenetics Laboratory, Department of Clinical Oncology, State Key Laboratory of Translational Oncology, Sir YK Pao Center for Cancer and Li Ka Shing Institute of Health Sciences, The Chinese University of Hong Kong, Shatin, Hong Kong

**Keywords:** *ZBTB16*, Methylation, *BCL6*, *ZBTB28*, Breast cancer

## Abstract

**Background:**

Breast cancer remains in urgent need of reliable diagnostic and prognostic markers. Zinc finger and BTB/POZ domain-containing family proteins (ZBTBs) are important transcription factors functioning as oncogenes or tumor suppressors. The role and regulation of ZBTB16 in breast cancer remain to be established.

**Methods:**

Reverse-transcription PCR and methylation-specific PCR were applied to detect expression and methylation of *ZBTB16* in breast cancer cell lines and tissues. The effects of ZBTB16 in breast cancer cells were examined via cell viability, CCK8, Transwell, colony formation, and flow cytometric assays. Xenografts and immunohistochemistry analyses were conducted to determine the effects of ZBTB16 on tumorigenesis in vivo. The specific mechanisms of ZBTB16 were further investigated using Western blot, qRT-PCR, luciferase assay, and co-IP.

**Results:**

*ZBTB16* was frequently downregulated in breast cancer cell lines in correlation with its promoter CpG methylation status. Restoration of ZBTB16 expression led to induction of G2/M phase arrest and apoptosis, inhibition of migration and invasion, reversal of EMT, and suppression of cell proliferation, both in vitro and in vivo. Furthermore, ectopically expressed ZBTB16 formed heterodimers with ZBTB28 or BCL6/ZBTB27 and exerted tumor suppressor effects through upregulation of ZBTB28 and antagonistic activity on BCL6.

**Conclusions:**

Low expression of *ZBTB16* is associated with its promoter hypermethylation and restoration of ZBTB16 inhibits tumorigenesis. ZBTB16 functions as a tumor suppressor through upregulating ZBTB28 and antagonizing BCL6. Our findings also support the possibility of ZBTB16 being a prognostic biomarker for breast cancer.

## Background

Breast cancer is one of the most common malignancies in women worldwide [1–4], with carcinomas arising from the epithelial component of the breast accounting for more than 99% cases [5]. In 2014, about 278,900 new cases were reported in China alone, representing the highest incidence of female malignant tumors [6]. The occurrence and development of breast cancer is affected by numerous factors, including gene mutations particularly in *BRCA1* or *BRCA2* [7–10] and epigenetic events [11] such as non-coding RNAs [12]. Surgery remains the primary treatment option [13–15], but has not seen much development for some time. In the meantime, advances in non-surgical therapeutic options have greatly improved the survival rates of patients, with a recorded 7.3% increase in the 5-year survival rate in China from 2000 to 2014 [16]. Despite significant progress in treatment strategies over recent years, prognosis of some breast cancer types, in particular, triple-negative breast cancer, remains unsatisfactory [4, 5, 7–10]. In-depth research on the mechanisms underlying etiology and pathogenesis should facilitate the identification of more reliable diagnostic and prognostic markers that may assist in the development of novel targeted therapeutic drugs to improve patient outcomes.

Zinc finger and BTB domain-containing 16 (*ZBTB16*), also known as promyelocytic leukemia zinc finger protein (*PLZF*) or zinc finger protein 145 (*ZFP145*), is a protein-coding gene initially discovered in a patient with acute promyelocytic leukemia (APL) in 1993 with a t(11;17) reciprocal chromosomal translocation resulting in an in-frame fusion with the retinoic acid receptor alpha (*RARα*) gene. The PLZF-RAR[α] fusion protein suppresses the normal functions of PLZF and RARα and is implicated in APL development [17]. *ZBTB16* is located on chromosome 11q23 and belongs to the zinc finger and BTB/POZ (poxvirus and zinc finger) domain-containing protein (ZBTB) family. The gene has three transcripts (Ensembl Gene ID:ENST00000335953, ENST00000392996, ENST00000310883), all encoding functional proteins. The translated protein contains nine C-terminal Krüppel-type sequence-specific zinc finger domains, an N-terminal BTB/POZ domain, and three portions of the RD2 subdomains. The C-terminal zinc finger domains promote binding of sequence-specific DNA to its target gene to perform a transcriptional repressor role [18]. N-terminal BTB/POZ domains function in DNA cycling, protein dimerization transformation, and protein/protein interactions to form multi-protein complexes that play physiologically significant roles [19, 20]. The RD2 domain is less well characterized than the BTB/POZ domain although mutations in this region have been shown to affect the transcriptional activity of *ZBTB16* [21]. ZBTB16 is widely expressed in various normal tissues, including CNS cells, hematopoietic cells, respiratory epithelial cells, cardiac muscle, and skeletal muscle cells [22, 23]. The protein is required for maintaining the self-renewal ability of early progenitor and spermatogonial cells [24, 25]. Previous reports have shown that this gene is under-expressed or silenced in multiple tumor tissues or corresponding cells in various cancer types, such as prostate cancer [26], primary malignant melanoma tumors [27], highly invasive malignant mesothelioma [28], hepatocellular carcinoma [29], lung cancer [30, 31], pancreatic cancer, and thyroid carcinoma [32, 33]. However, the specific role of ZBTB16 in breast cancer remains to be established.

ZBTB27 (BCL6) and ZBTB28 (BCL6B) are also members of the ZBTB family. *BCL6* has been characterized as an oncogene and *ZBTB28* acts as a tumor suppressor gene (TSG) in breast cancer. Our previous study has demonstrated that BCL6 and ZBTB28 are co-localized and form a complex in multiple tumor cell lines. Both proteins contain highly similar zinc finger structure and thus could competitively regulate p53 expression, while BCL6 itself is a direct target gene of ZBTB28 [34]. Co-localization and heterodimerization between ZBTB16 and BCL6 that display high structural homology have additionally been validated [35]. However, the specific associations among ZBTB16, BCL6, and ZBTB28 and their relevance in breast cancer remain to be established.

We observed lower *ZBTB16* expression in breast cancer relative to normal tissue samples, which was attributed to gene silencing via methylation of the promoter region. Decreased gene expression was positively associated with poor prognosis in breast cancer patients. Upon restoration of expression, ZBTB16 inhibited proliferation and metastasis, induced apoptosis, and blocked the cell cycle progression in breast cancer cells. Moreover, our study revealed associations among ZBTB16, BCL6, and ZBTB28 for the first time. The collective results demonstrated that ZBTB16 exerts significant tumor suppressor effects through upregulation of *ZBTB28* and downregulation of *BCL6* and supported its identity as a prognostic biomarker to improve treatment outcomes of breast cancer.

## Results

### ZBTB16 is downregulated in breast cancer and associated with prognosis

**Fig. 1:**
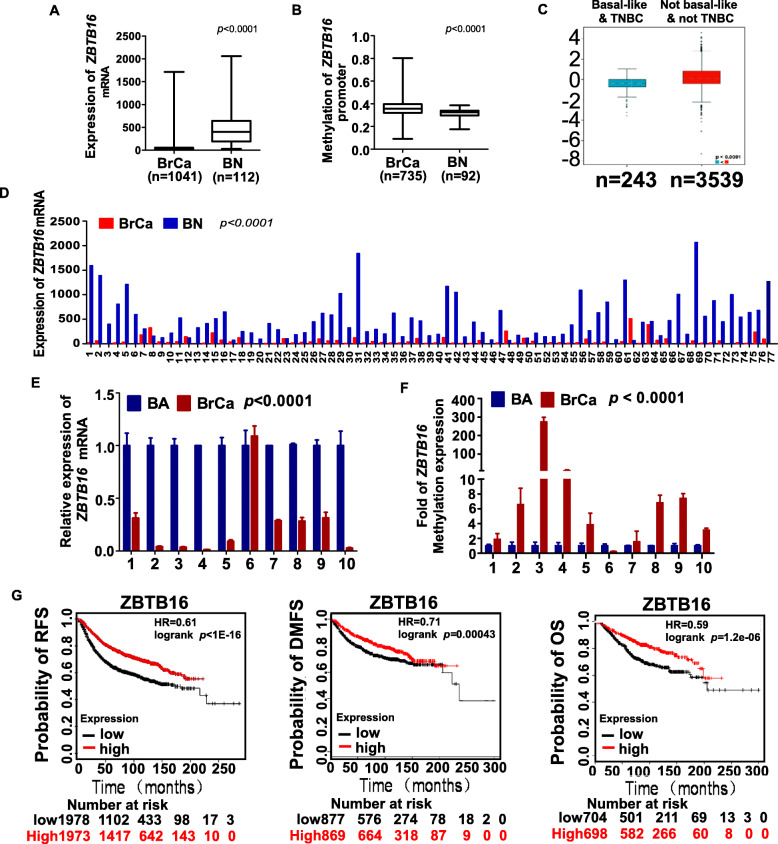
Expression and methylation of *ZBTB16* in database. **a** The expression of *ZBTB16* in 1041 breast tumor specimens and 112 normal breast tissue samples in TCGA. *p* value was assessed by Mann-Whitney *U* test. **b** The methylation status of *ZBTB16* in 735 breast cancer specimens and 92 normal breast tissue samples in TCGA. *p* value was assessed by Mann-Whitney *U* test. **c** The expression of *ZBTB16* in triple-negative breast cancer (TNBC) and basal-like breast cancer (*n* = 243) and not basal-like BrCa and not TNBC group (*n* = 3539). **d***ZBTB16* expression in 77 pairs of breast cancer specimens and normal breast tissues in MethCH. **e**, **f** The expression and methylation level of *ZBTB16* in 10 breast cancer tissues compared with paired non-cancerous breast tissues. **g** The higher *ZBTB16* expression was association with better patient survival detected in the Kaplan-Meier Plotter dataset

To explore the significance of ZBTB16 in breast cancer, we analyzed the expression of ZBTB16 in 1041 breast tumor and 112 normal breast tissue samples in TCGA along with its promoter methylation status in 735 breast cancer and 92 normal breast tissue samples. In breast cancer specimens, *ZBTB16* expression was significantly downregulated with increased promoter methylation (Fig. 1a, b). Furthermore, in triple-negative breast cancer (TNBC) and basal-like breast cancer (*n* = 243) cases, *ZBTB16* expression was lower than that in non-basal-like breast cancer and non-TNBC groups (*n* = 3539) in bc-GenExMiner (Fig. 1c). MethHC data (http://methhc.mbc.nctu. edu.tw/php/index.php) showed that *ZBTB16* was downregulated in 77 paired breast cancer tissues (Fig. 1d). Abnormal methylation of promoters of tumor suppressor genes induces gene inactivation, which is critical in pathogenesis of almost all human tumors. Our data also demonstrated *ZBTB16* methylation was significantly increased while the expression of *ZBTB16* was sharply decreased in 10 breast cancer tissues compared with paired non-cancerous breast tissues (Fig. 1e, f). Importantly, higher *ZBTB16* expression was associated with greater survival rates, as determined from the Kaplan-Meier Plotter dataset (http://kmplot.com /analysis/). Conversely, low *ZBTB16* expression was correlated with poorer relapse-free survival (RFS), distant metastasis-free survival (DMFS), and overall survival (OS) (Fig. 1g). Based on the data, we proposed that *ZBTB16* is a tumor suppressor downregulated in breast cancer, possibly due to abnormal promoter methylation.

### ZBTB16 downregulation in breast cancer occurs via promoter hypermethylation

**Fig. 2:**
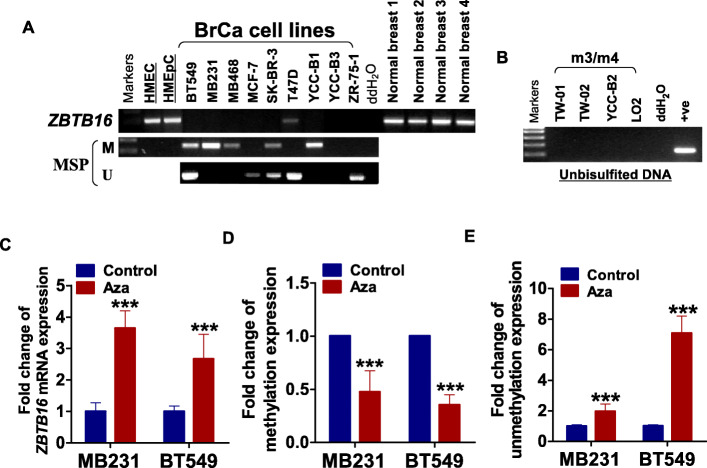
Methylation and demethylation of *ZBTB16* in breast cancer cells and tissues. **a***ZBTB16* expression and methylation status in breast cancer cell lines. **b** The specificity of methylated primers was confirmed by not-bisulfite DNA. **c** Detection of ZBTB16 expression by RT-PCR before and after Aza treatment in breast tumor cells. **d**, **e** Methylation and unmethylation level before and after drug treatment in breast cancer cell lines. Methylation and demethylation were confirmed by quantitative methylation-specific PCR (qMSP). **p < 0.01; ***p < 0.001

RT-PCR analysis showed *ZBTB16* expressed in human mammary epithelial cell HMEC and MCF10A, as well as normal adult breast tissues and other human normal tissues (sFig1A), but downregulated or silenced in nine breast cancer cell lines. MSP analysis disclosed *ZBTB16* promoter methylation was present in more than half of the breast cancer cell lines, where ZBTB16 expression was downregulated or depleted (Fig. 2a). The specificity of methylated primers was confirmed by not-bisulfite DNA (Fig. 2b). After treatment with the DNA methyltransferase inhibitor 5-aza-2′-deoxycytidine (Aza), expression of *ZBTB16* was restored, concomitant with decreased methylation levels (Fig. 2c–e). Our collective preliminary findings supported *ZBTB16* is inactivated via promoter hypermethylation in breast cancer.

### ZBTB16 methylation and its clinical correlations in breast cancer

To address whether methylation occurs in primary tumors, we analyzed the methylation status of *ZBTB16* in 152 breast tumor samples and 16 normal breast tissues using MSP. *ZBTB16* promoter methylation was observed in 79 of 152 (51.9%) primary tumors and 0 of 16 (0%) normal breast tissues (Table 1). We further analyzed the correlations of *ZBTB16* methylation with clinicopathological features (Table 2). However, there was no association of *ZBTB16* methylation with clinicopathological characteristics of patients, including age, tumor grade and size, lymph node metastasis, distant metastasis, *ER*, *PR*, *HER2*, or *p53* status.

**Table 1:** Methylation status of *ZBTB16* promoter in primary breast tumors

Samples	*ZBTB16* promoter		Frequency of methylation
	Methylation	Unmethylation	
BrCa (*n* = 152)	79	73	79/152 (51.9%)
BN (*n* = 16)	0	16	0/16 (0%)

Note: *BrCa* breast cancer, *BN* breast normal tissues

**Table 2:** *ZBTB16 *methylation and clinicopathological features of breast tumors

Clinicopathological features	Number (***n*** = 152)	Methylated status		***p*** value
		Unmethylated	Methylated	
**Age**				
≤ 40	24	12	12	0.833
> 40	128	67	61	
**Grade**				
I	10	3	7	0.123
II	102	50	52	
III	9	7	2	
Unknown	31	19	12	
**Tumor size**				
< 2.0 cm	43	17	26	0.277
≥ 2.0 cm, <5.0 cm	85	49	36	
≥ 5.0 cm	18	10	8	
Unknown	6	3	3	
**Lymph node metastasis**				
Positive	72	39	33	0.664
Negative	72	37	35	
Unknown	8	3	5	
**ER**				
Positive	84	43	41	0.977
Negative	51	27	24	
Unknown	17	9	8	
**PR**				
Positive	67	37	30	0.653
Negative	67	32	35	
Unknown	18	10	8	
**HER2**				
Positive	107	54	53	0.736
Negative	25	13	12	
Unknown	20	12	8	
**Distant metastasis**				
Positive	2	0	2	0.072
Negative	139	76	63	
Unknown	11	3	8	
**p53**				
Positive	64	32	32	0.574
Negative	51	25	26	
Unknown	37	22	15	

### ZBTB16 is a functional tumor suppressor in breast cancer

**Fig. 3:**
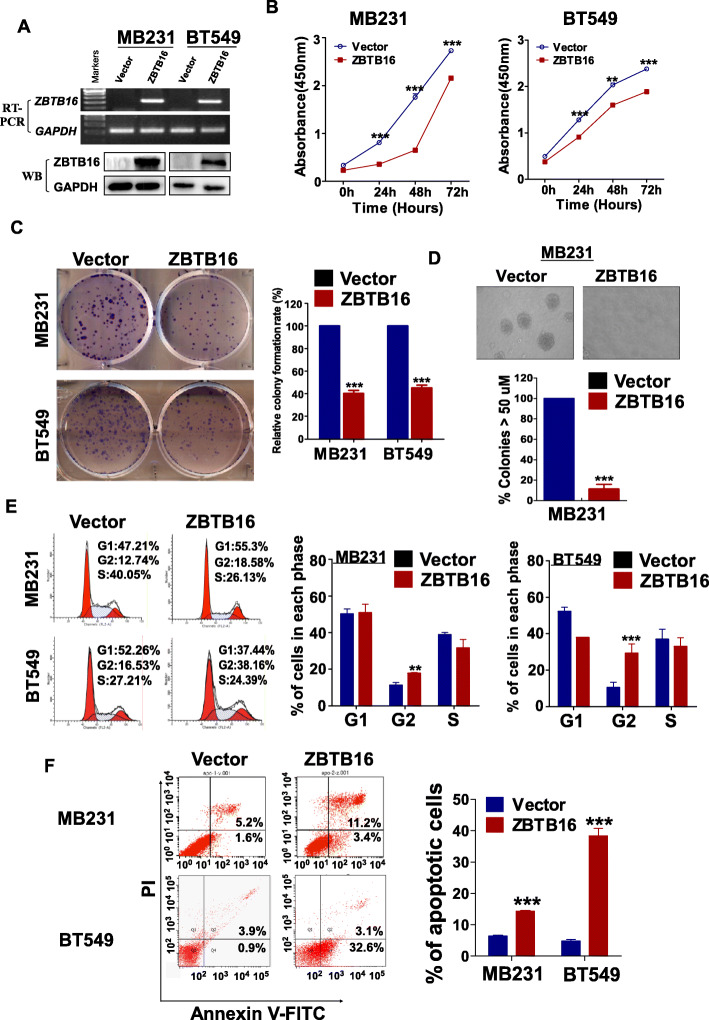
ZBTB16 inhibits breast cancer cells growth in vitro. **a** Detection of ZBTB16 overexpression by RT-PCR (upper panel) and Western blot (lower panel), with GAPDH as a control. Ectopic expression of ZBTB16 inhibited carcinoma cell growth, as assessed by CCK8 (**b**), colony formation (**c**), or soft agar assays (**d**).Values were shown as mean ± standard error from three independent experiments. **e** Cell cycle distribution of vector- or ZBTB16-transfected cells as determined by flow cytometry. Representative flow cytometry plots. Histograms of cell cycle alterations. f Induction of apoptosis detected by flow cytometry. Histograms of apoptosis rate were shown at the right. Values were shown as mean ± standard error from three independent experiments. **p < 0.01; ***p < 0.001

To reveal functions of ZBTB16 in BrCa (breast cancer), firstly, we examined the effect of ZBTB16 on cell proliferation activity. Specifically, MDA-MB-231 and BT549 human breast cancer cell lines were stably transfected with pcDNA3.1 or ZBTB16- expressing plasmids and mRNA and protein expression of ZBTB16 were detected via RT-PCR and Western blot, respectively (Fig. 3a). The effects of ZBTB16 on cell proliferation and viability were further examined via CCK8 and colony formation assays. ZBTB16 overexpression was significantly associated with inhibition of cell proliferation and viability (Fig. 3b–d). Subsequent flow cytometry analysis showed that ZBTB16-overexpressing cells comprised a significantly higher proportion of cells in the G2-M phase, compared to the control cell group (Fig. 3e). Higher spontaneous apoptosis rates of ZBTB16- overexpressing cells were detected via flow cytometry with Annexin V-FITC and PI staining (Fig. 3f). These results suggested that ZBTB16 exerts an anti-tumor effect through inhibition of cell proliferation and induction of cell cycle arrest and apoptosis in breast cancer cells.

### ZBTB16 inhibits breast cancer cell migration and invasion by reversing EMT

**Fig. 4:**
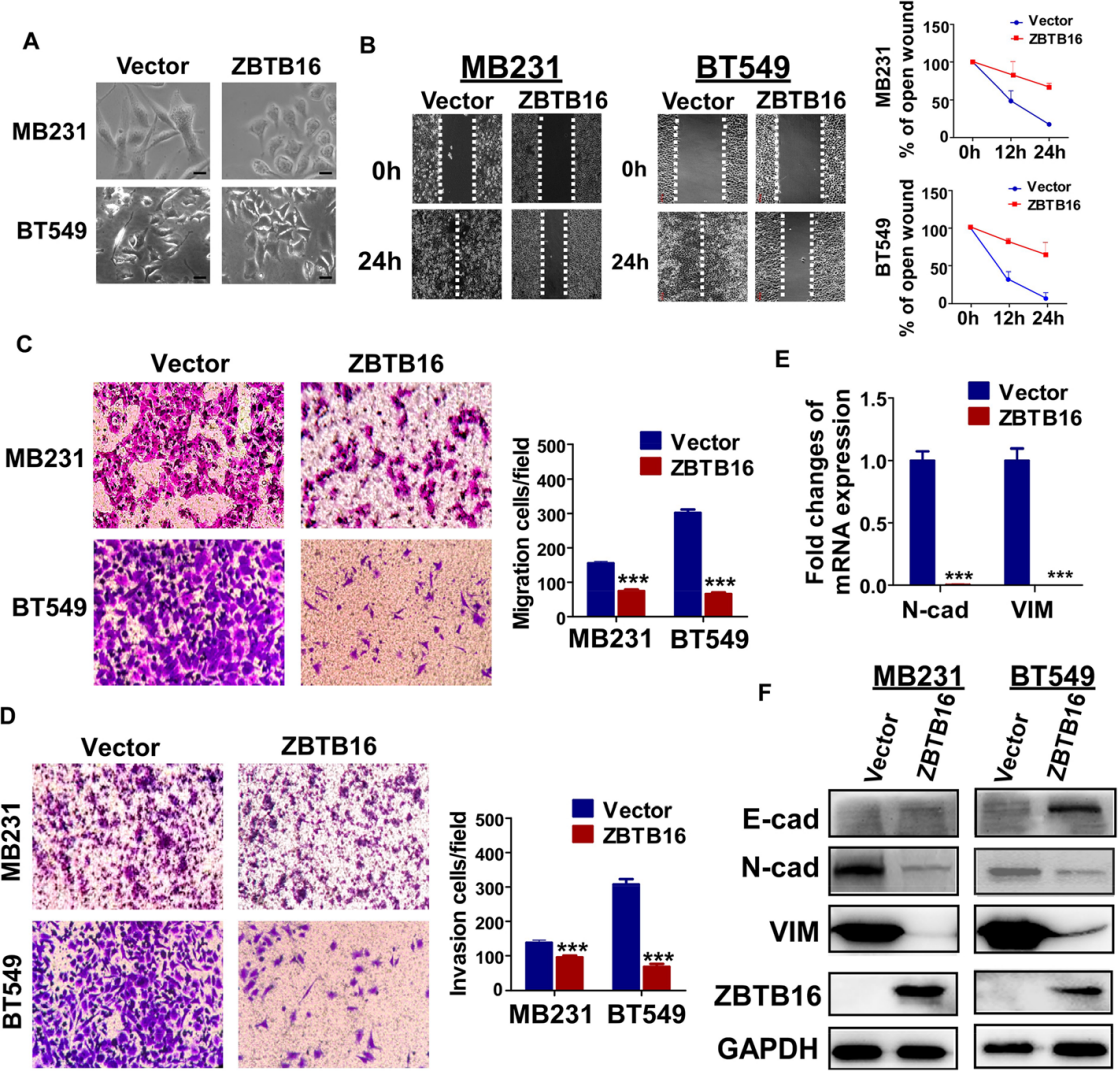
*ZBTB16* expression inhibits the migration and invasion of carcinoma cells through reversing EMT. **a** Morphological changes of cells transfected with ZBTB16 or empty vector, obtained by phase contrast microscopy. Original magnification, × 400. **b** Representative image of wound healing assay in vector- and ZBTB16-expressed MB231 and BT549 cells. **c** Representative image (left) and the histogram statistics (right) of Transwell cell migration assay in vector- and ZBTB16-expressing MB231 and BT549 cells, × 100 magnification (*p* < 0.001). *p* value was assessed by *t* test. **d** Representative image (left) and the histogram statistics (right) of Transwell cell invasion assay in vector- and ZBTB16-expressing MB231 and BT549 cells, × 100 magnification (*p* < 0.001). *p* value was assessed by *t* test. **e** Expression of EMT markers in ZBTB16-expressing carcinoma cells, as determined by qRT-PCR. VIM:Vimentin (**f**) and Western blot, GAPDH was used as a control. **p < 0.01; ***p < 0.001

We further focused on the potential effect of ZBTB16 on metastasis. ZBTB16 overexpression resulted in partial morphological changes from scattered growth structures to tightly packed colonies (Fig. 4a). The results of wound healing assays showed that over 24 h, the scratch healing rate of confluent layers of MDA-MB231 and BT549 cell lines overexpressing ZBTB16 was significantly slower than that of control cell layers (Fig. 4b). In Transwell assay, the number of ZBTB16-expressing cells passing through the chamber was significantly lower than that in the control group (Fig. 4c). Furthermore, in Transwell assays, including a Matrigel barrier, the number of breast cancer cells with ZBTB16 overexpression invading the barrier was markedly decreased relative to control cells (Fig. 4d). Our collective results suggested that ZBTB16 effectively suppresses migration and invasion activities of breast cancer cells. Epithelial-mesenchymal transition (EMT) is well established as a critical step of invasion and metastasis. Data analysis of the GCE database also revealed correlations between ZBTB16 and EMT markers (sFig2). In qRT-PCR analyses, ectopic ZBTB16 induced significant downregulation of *N-cadherin* and *Vimentin* mRNA in MDA-MB231 cell lines (Fig. 4e). Simultaneously, Western blot analyses showed that ectopic ZBTB16 induced an increase in the epithelial marker, E-cadherin, and suppressed expression of the mesenchymal markers, N-cadherin and Vimentin, at the protein level (Fig. 4f). Based on these findings, we proposed that ZBTB16 inhibits migration, invasion, and reverses EMT in breast cancer cells.

### ZBTB16 suppresses xenograft breast tumor growth in nude mice

**Fig. 5:**
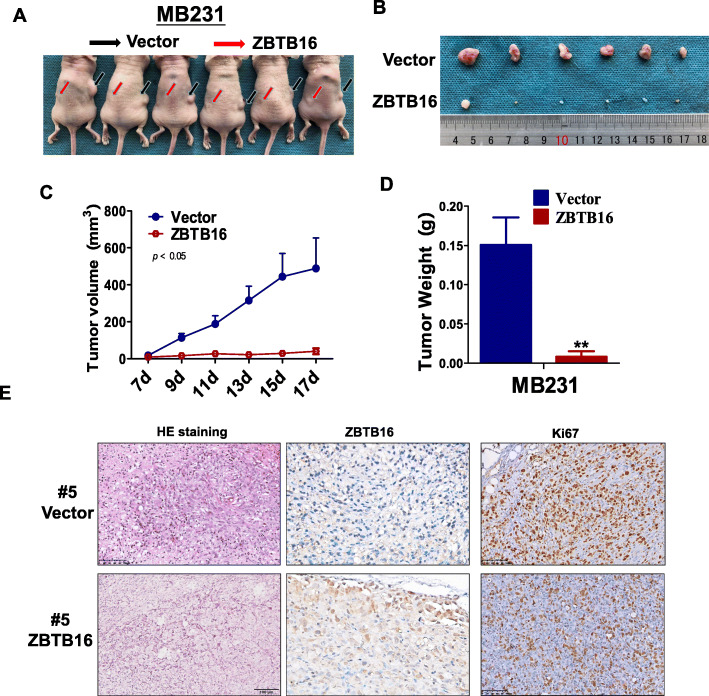
*ZBTB16* suppresses xenograft breast tumor growth in nude mice. **a** Images of human BrCa tumor xenograft. Red and black arrows indicated empty vector control and ZBTB16-overexpressing tumors. **b** Tumor tissues from empty vector control and ZBTB16-overexpressing tumors (*n* = 6). **c** Growth curve of xenograft tumors. Tumor volume was calculated from tumor length and width, measured every two days. *p* value was assessed by *t* test. **d** Tumor weight measurements from empty vector control and ZBTB16-overexpressing tumors (*n* = 6). *p* value was assessed by *t* test. **p < 0.01. **e** Immunohistochemistry (IHC) and HE staining were used to evaluate the proliferation and nuclear fragmentation levels in nude mouse tissues

The above experiments confirmed the tumor suppressor activity of ZBTB16 in vitro. The nude mouse xenograft tumor model was subsequently employed to examine the anti-tumor ability of ZBTB16 in vivo. Mean weight and volume of tumors were significantly lower in mice that received ZBTB16-transfected cells, compared to the control group (Fig. 5a–d). Immunohistochemistry (IHC) and HE staining showed a significant decrease in the proliferation index in nude mouse xenograft tumor tissues of ZBTB16-transfected cells (Fig. 5e). These data indicated that ZBTB16 has the capability to significantly impede tumor growth, both in vitro and in vivo, validating an important role of ZBTB16 as a tumor suppressor gene in breast cancer.

### ZBTB16 enhances the promoter activity of ZBTB28 and suppresses that of BCL6 in breast cancer cells

ZBTB16, BCL6, and ZBTB28 are members of the ZBTB family. Notably, *BCL6* acts as an oncogene while *ZBTB28* is a TSG. Furthermore, BCL6 has been identified as a direct repressional target of ZBTB28 based on colocalization and heteromerization between the two proteins [34]. Here, we explored the associations among ZBTB16, BCL6, and ZBTB28 and their relevance in breast cancer. Expression of ZBTB16 and ZBTB28 in most breast cancer cell lines was low or silenced but BCL6 was high. This data was obtained from TCGA cancer dataset and accessed through cBioPortal (www.cbioportal.org) (sFig 1B).

**Fig. 6:**
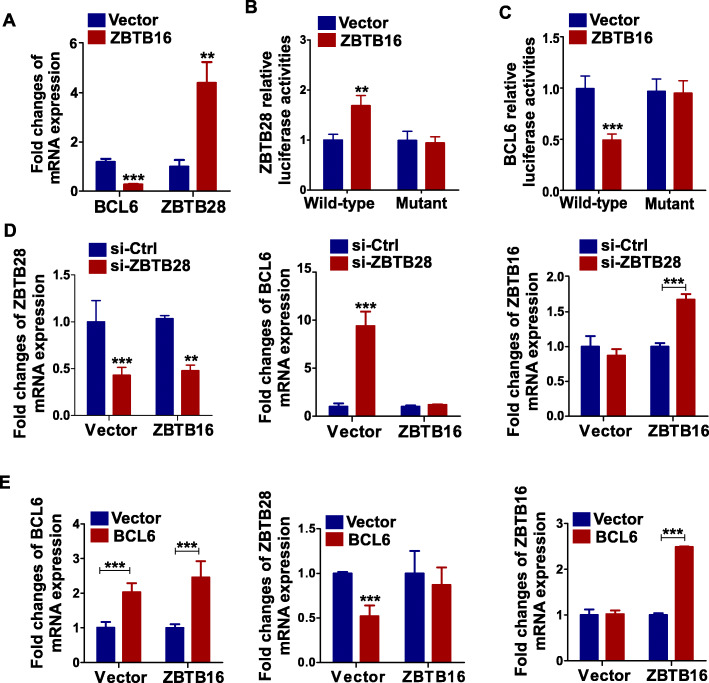
ZBTB16 regulates ZBTB28 and BCL6. **a** Expression of *ZBTB28* and *BCL6* in *ZBTB16* stably expression BT549 cells by qRT-PCR. **b**, **c** ZBTB16 promoted *ZBTB28* and inhibited *BCL6* promoter activities, as detected by dual luciferase reporter system. **d** Expression of *ZBTB28*, *BCL6*, and *ZBTB16* after si-*ZBTB28* in vector-BT549 and ZBTB16-BT549 cells. **e** Expression of *ZBTB28*, *BCL6*, and *ZBTB16* after overexpression of *BCL6* in vector-BT549 and ZBTB16-BT549 cells. **p < 0.01; ***p < 0.001

Since wild-type MDA-MB-231 did not express ZBTB28, we selected the BT549 cell line for analysis. qRT-PCR experiments showed that compared with the control group, *BCL6* was downregulated while *ZBTB28* was upregulated in BT549 cells that stably express ZBTB16 (Fig. 6a). Moreover, the regulatory mechanisms of action of ZBTB16 on *ZBTB28* and *BCL6* were examined using the luciferase reporter system. Data from the luciferase assay showed that ZBTB16 suppressed the promoter activity of *BCL6* and enhanced that of *ZBTB28* (Fig. 6b, c), indicating that ZBTB16 might play an anti-tumor role in breast cancer through regulating ZBTB28 and BCL6.

Next, we used siRNA to eliminate ZBTB28. Specifically, control siRNA and siRNA-ZBTB28 were individually transfected into vector-BT549 and stable ZBTB16-BT549 cells and qRT-PCR was applied to examine the efficiency of knockdown (Fig. 6d). Indeed, in vector-BT549 cells, transfection of siRNA-ZBTB28 led to upregulation of *BCL6* mRNA relative to siRNA control, as described previously [34]. In contrast, *BCL6* expression levels were not altered in ZBTB16-BT549 cells. Simultaneously, in vector-BT549 cells, no differences in expression of ZBTB16 were observed between siRNA control and siRNA-ZBTB28 groups. However, transfection of siRNA-ZBTB28 into ZBTB16-BT549 cells induced upregulation of ZBTB16 (Fig. 6d). Based on the same theory, we generated a plasmid overexpressing BCL6 and subsequently transfected vector or BCL6 into vector-BT549 and stable ZBTB16-BT549 cells. The efficiency of overexpression was examined by qRT-PCR. As described previously, in vector-BT549 cells, ectopic expression of BCL6 led to downregulation of *ZBTB28* mRNA relative to vector. However, *ZBTB28* expression levels were not altered in ZBTB16-BT549 cells. Simultaneously, in vector-BT549 cells, no differences in expression of *ZBTB16* were observed between vector and BCL6 overexpression groups. However, ectopic expression of BCL6 induced upregulation of *ZBTB16* in ZBTB16-BT549 cells (Fig. 6e). In view of the above findings, we propose that ZBTB16 enhances the promoter activity of *ZBTB28* while suppressing that of *BCL6* in breast cancer cells. And ZBTB16 may exert anticancer effects via upregulating *ZBTB28* and downregulating *BCL6*.

### Antitumor effects of ZBTB16 partially depend on ZBTB28 and BCL6

**Fig. 7:**
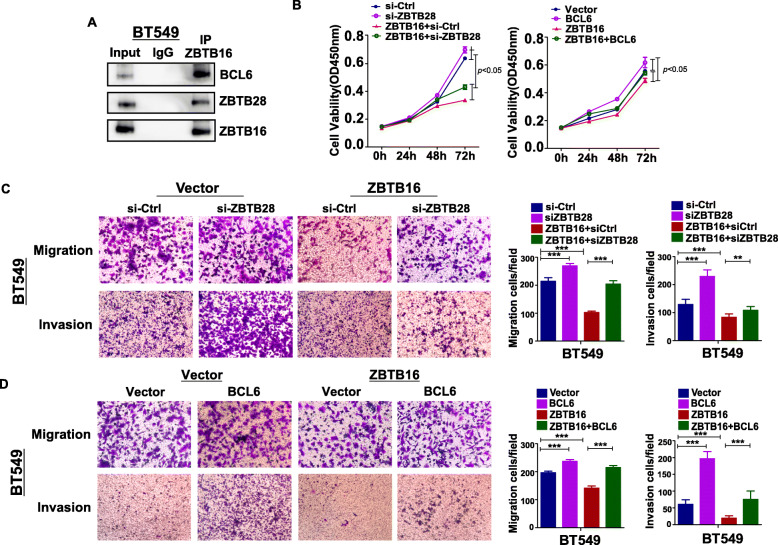
ZBTB16 forms heterodimer with ZBTB28 and BCL6, respectively, and upregulating *ZBTB28* and antagonizing *BCL6*. **a** Co-IP outcome revealed the binding between BCL6, ZBTB28, and ZBTB16. **b** Cell viability in si-*ZBTB28* or BCL6 overexpression group compared to si-control or vector group in vector-BT549 cell and ZBTB16-BT549 cell by CCK8 assay. **c**, **d** Cell migration and invasion ability in si-*ZBTB28* or BCL6 overexpression group compared to si-control or vector group in vector-BT549 cell and ZBTB16-BT549 cell by Transwell assay. *p* value was assessed by *t* test. **p < 0.01; ***p < 0.001

To further establish the underlying tumor suppression mechanisms of ZBTB16, Co-IP and replenishment experiments were performed. Co-IP experiments validated interaction of ZBTB16 with ZBTB28 and BCL6 (Fig. 7a). Replenishment experiments with ZBTB28 knockdown or BCL6 overexpression in vector-BT549 and ZBTB16-BT549 cells were conducted. As expected, in replenishment CCK8 assays, cell viability was higher in si-ZBTB28 and BCL6 overexpression groups, compared to si-control and vector groups, respectively, in vector-BT549 and ZBTB16-BT549 cells. Moreover, the overall viability of vector-BT549 cells was markedly higher than that of ZBTB16-BT549 cells (Fig. 7b). Since ZBTB16, ZBTB28, and BCL6 were associated with tumor metastasis, replenishment Transwell assays were used to confirm our hypothesis. Migration and invasion abilities of vector-BT549 and ZBTB16-BT549 cells were enhanced in the si-ZBTB28 and BCL6 overexpression groups, compared to si-control and vector groups. Furthermore, migration and invasion abilities of vector-BT549 cells were significantly higher than those of ZBTB16-BT549 cells (Fig. 7c, d). Overall, our results showed that ZBTB16 may form heterodimers with ZBTB28 or BCL6 and exert tumor suppressor effects in breast cancer through upregulation of *ZBTB28* and suppression of *BCL6*.

## Discussion

The increasing incidence of breast cancer is a global health threat for women. Identification of effective tumor markers for use in early diagnosis and treatment should aid in the development of novel targeted therapies, reduction of recurrence, and improvement of survival rates and patient outcomes. Our experiments clearly demonstrated that ZBTB16 expression was decreased in breast cancer tissues, compared with normal breast and matched paracancerous tissues. ZBTB16 overexpression led to inhibition of proliferation, migration, and invasion of breast cancer cells, reversal of the EMT process, blockage of the cell cycle in G2-M phase, and apoptosis of breast cancer cells in vitro. In addition, upon transplantation of breast cancer cells that stably express ZBTB16 into nude mice in vivo, tumor sizes, and weights, along with Ki-67 expression, were significantly decreased.

The POZ and Kruppel (POK) transcription factor family of proteins play significant roles in protein dimerization transformation and protein-protein interactions to form multi-protein complexes through the N-terminal shared BTB domain [19, 20]. The C-terminal shared C2H2 zinc finger domain promotes binding of sequence-specific DNA to its target gene [18]. The transcription factors of POK family form homodimers with itself or heterodimers with other POK proteins, which are recruited to the binding sites of downstream target genes and exert gene regulatory effects [35–39]. A study has reported that ZBTB16 is involved in the development of promyelocytic leukemia through interactions of the BTB-POZ domain with BCL6 [40]. Further analyses have disclosed that BCL6 and ZBTB16 interact directly with each other, facilitating simultaneous recruitment in polyprotein nuclear complexes and co-localization in nuclei of multiple cell lines [35]. Our previous study has revealed that ZBTB28 and BCL6 are known to co-localize and form complexes in the nuclei of several cell types [34]. Under normal condition endogenous BCL6 and ZBTB16 are co-induced and partially colocalized in myeloid MDS cells, COS6 (African green monkey kidney cells), or CHO (Chinese Hamster Ovary, CHO) cells, indicating that BCL6 and ZBTB16 may interact physiologically. In present study, we did not detect these interactions in normal cells. Further investigations need to be conducted to reveal the detailed mechanism.

**Fig. 8:**
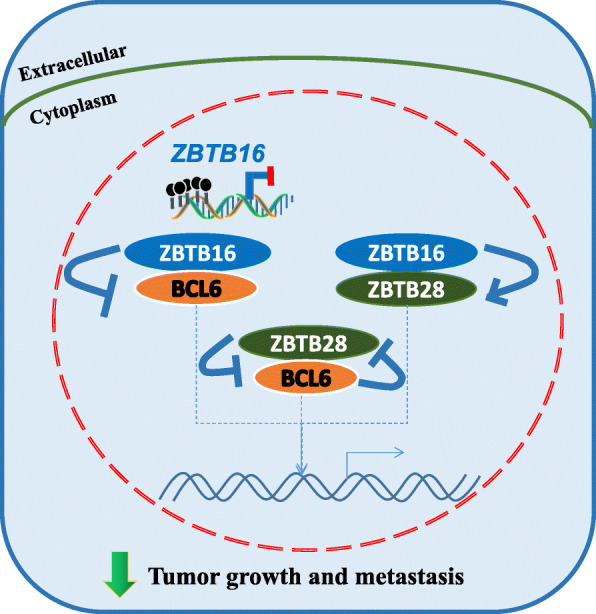
A scheme of ZBTB16 effects on ZBTB28 and BCL6. ZBTB16 may form complexes with ZBTB28 and BCL6, respectively, and exert tumor suppressor effects through upregulation of ZBTB28 and antagonistic activity on BCL6 in breast cancer

Based on our data, we presented a hypothetical model that ZBTB16 might form complexes with ZBTB28 and BCL6, respectively, and function as a tumor suppressor through inhibition of BCL6 and promotion of ZBTB28 (Fig. 8). Different studies suggested a proto-oncogene role for BCL6 and a tumor suppressor role for ZBTB28. Indeed, our previous study has indicated that ZBTB28 acts as a tumor suppressor in multiple cancer cells by inhibiting BCL6 expression and promoting p53 expression. In present study, however, knockdown of ZBTB28 did not upregulate BCL6 expression, while overexpressed BCL6 did not inhibit ZBTB28 expression in cell lines stably expressing ZBTB16, indicating a disruption of reciprocal inhibition between BCL6 and ZBTB28 in cells with ZBTB16 expression. More importantly, ZBTB16 was upregulated in these cells with knockdown of ZBTB28 or overexpression of BCL6. However, no ZBTB16 changes were detected in cells with ZBTB16 silence regardless of knockdown of ZBTB28 or overexpression of BCL6. These findings, in combination with luciferase and other experimental results, lead to a reasonable assumption that since ZBTB16 suppresses the promoter activity of BCL6 and enhances that of ZBTB28, ZBTB16 expression increases during feedback regulation under conditions of ZBTB28 knockdown or BCL6 overexpression, in turn, further inhibiting expression of BCL6, which results in no overall expression changes in cell lines stably expressing ZBTB16. This phenomenon does not occur in control cell lines with ZBTB16 silence. Another theory worth exploring is that ZBTB28 and BCL6 may compete to bind to target p53 for regulation of gene expression in a heterodimeric form to exert anti-cancer effects [34]. The *PLZF-RARα* gene formed by fusion of ZBTB16 with the retinoic acid receptor alpha (*RARα*) gene may act as a *p53* gene competitive transcriptional repressor [41]. Accordingly, we propose that ZBTB16, ZBTB28, and BCL6 may mutually regulate gene expression through the *p53* pathway via heterodimeric interactions.

Abnormal gene function and cellular pathways caused by epigenetic events, such as DNA methylation and histone acetylation, are clearly associated with human cancers [42]. In the cancer microenvironment, inactivation of tumor suppressor genes via promoter methylation contributes to tumor occurrence, development, and metastasis. The promoter region of the *ZBTB16* gene is rich in CpG islands, supporting the conclusion that methylation in this region inhibits gene expression. Our data showed that 51.9% of breast cancer tissues were methylated, while no methylation was detected in normal breast tissues, indicating that the promoter methylation of ZBTB16 is a frequent and tumor-specific event in breast cancer. The collective findings of this study suggested that *ZBTB16* may be effectively used as a biomarker to improve prognosis and treatment of breast cancer patients.

## Materials and methods

### Cell lines, tumor samples, and normal tissues

Multiple cancer cell lines (MDA-MB-231, BT549) were obtained from the American Type Culture Collection (ATCC, Manassas, VA, USA) or collaborators and maintained in RPMI 1640 medium (Gibco-BRL, Karlsruhe, Germany) with 10% fetal bovine serum (FBS) and 1% penicillin and streptomycin at 37°C/5% CO_2_, as recommended by ATCC. All tissue samples used, including primary tumor and paired surgical margin tissues as well as normal tissues, were obtained from the First Affiliated Hospital of Chongqing Medical University. Tissue samples were pathologically and histologically examined to ensure that the percentage of tumor cells was > 70%, followed by collection of clinical and pathological data. This study was authorized by the Institutional Ethics Committees of the First Affiliated Hospital of Chongqing Medical University (Approval notice: # 2016-75) and abided by the Declaration of Helsinki.

### Expression, prognosis, and methylation data processing

Clinical information and methylation data were accessed and downloaded from The Cancer Genome Atlas (TCGA) data portal. In the TCGA invasive breast cancer (BRCA) data set, there were 1041 BrCa (breast cancer) samples and 112 BN (breast normal) samples. In which, 735 BrCa samples and 92 BN samples were detected methylation status. Sequences of cDNA were analyzed using the NCBI Blast program (http://www.ncbi.nlm. nih.gov/BLAST/). Basic information on the ZBTB16 gene was obtained from GeneCards (https://www.genecards.org/). Expression and methylation data on ZBTB16 in normal and breast cancer tissues were acquired from ATCC (https://www.atcc.org/en.aspx) and MethHC (http://methhc.mbc.nctu. edu.tw/php/index.php). Survival analysis of breast cancer patients in association with ZBTB16 expression was conducted using KM plotter (http://kmplot.com/ analysis/index).

### RNA isolation, reverse transcription (RT)-PCR, and real-time PCR

Total RNA was isolated from cell lines and tissues using TRIzol Reagent® (Molecular Research Center, Cincinnati, OH, USA), according to the manufacturer’s instructions. Reverse transcriptase-polymerase chain reaction (RT-PCR) was performed on aliquots containing 1 μg total RNA to generate 20 μl cDNA. Real-time PCR was performed using Go-Taq (Promega, Madison, WI, USA) under the following conditions: initial denaturation at 95°C for 2 min, followed by 32 cycles (95°C for 30s, 55°C for 30s, and 72°C for 30s) of amplification, and final extension at 72°C for 3 min. β-actin was amplified as a control and a total of 23 PCR cycles performed. The primer sequences are shown in Table 3. Real-time quantitative PCR was performed with a SYBR Green kit (Thermo Fisher). All analyses were conducted using the 7500 Real-Time PCR System (Applied Biosystems, Foster City, CA, USA). Each sample was examined in triplicate. ß-actin was used as a control. Gene expression level was calculated by the 2−ΔΔCt method.

**Table 3:** List of primers used in this study

PCR	Primer	Sequence (5′-3′)	Product size (bp)	PCR cycles	Annealing temperature (°C)
**RT-PCR**	ZBTB16F	**GTCTCCATGGACTTCAGCAC**	**206 bp**	**32**	**55**
	ZBTB16R	**TACGTCTTCATCCCACTGTG**			
	β-actinF	**TCCTGTGGCATCCACGAAACT**	**315 bp**	**23**	**55**
	β-actinR	**GAAGCATTTGCGGTGGACGAT**			
**qRT-PCR**	ZBTB16F	**GTCTCCATGGACTTCAGCAC**	**206 bp**		**60**
	ZBTB16R	**TACGTCTTCATCCCACTGTG**			
	VIMF	**GACCAGCTAACCAACGACAA**	**150 bp**		**60**
	VIMR	**GTCAACATCCTGTCTGAAAGAT**			
	CDH2F	**CGAATGGATGAAAGACCCATCC**	**174 bp**		**60**
	CDH2R	**GGAGCCACTGCCTTCATAGTCAA**			
	BCL6F	**GACTTCATGTACACATCTCGGC**	**189 bp**		**60**
	BCL6R	**CATCAGCATCCGGCTGTTGA**			
	ZBTB28F	**CTACGTCCGCGAGTTCACTC**	**170 bp**		**60**
	ZBTB28R	**CCCGGAAAATTGAATAGAAG**			
	β-actinF	**GTCTTCCCCTCCATCGTG**	**113 bp**		**60**
	β-actinR	**AGGGTGAGGATGCCTCTCTT**			
**MSP**	ZBTB16m3	**CGGTGATATCGGAGTTCGTC**	**139 bp**	**40**	**60**
	ZBTB16m4	**GCGTACGAAAATATACGAAACG**			
	ZBTB16u3	**GTGGTGATATTGGAGTTTGTT**	**142 bp**	**40**	**58**
	ZBTB16u4	**ACACATACAAAAATATACAAAACA**			

### Bisulfite treatment and methylation-specific PCR (MSP)

Quantitative MSP (qMSP) was performed as described previously [43]. MSP amplification of bisulfite-treated DNA was performed at an annealing temperature of 60 °C for 40 cycles using the primers shown in Table 3. The MSP reaction was conducted using AmpliTaq-Gold DNA Polymerase (Applied Biosystems, Foster City, CA, USA) and qMSP with the 7500 Real-Time PCR System (Applied Biosystems). PCR products were analyzed using 2% agarose gel electrophoresis.

### Construction of vector- and ZBTB16-expressing stable cell lines

A ZBTB16-expressing stable cell line was generated. First, a ZBTB16-expressing plasmid was constructed by inserting *ZBTB16* full-length gene with a flag into pcDNA3.1(+) framework. The recombinant plasmid was sequenced. Next, pcDNA3.1 and ZBTB16-containing plasmid (4 μg) were transfected with Lipofectamine 2000 (Invitrogen, Carlsbad, USA) into MDA-MB-231 and BT549 cell lines. Forty-eight hours after transfection, G418 (Amresco, Solon, OH, USA) was added to the cells (1200 μg/ml for MDA-MB-231 and 200 μg/ml for BT549). Stably transfected ZBTB16 and control cell lines were obtained by continuous screening of G418 for 2 weeks. Ectopic expression of ZBTB16 was assessed via RT-PCR and Western blot prior to experiments.

### Cell proliferation and colony formation assays

MDA-MB-231 and BT549 cells were cultured in 96-well plates at a density of 2000 cells/well after transfection with ZBTB16-expressing or control (pcDNA3.1) plasmids. Cell proliferation was measured at 0, 24, 48, and 72 h using Cell Counting Kit-8 (CCK-8; Beyotime, Shanghai, China) and absorbance read on a microplate reader at 450 nm. The colony formation assay (CFA) was additionally used to detect cell proliferation. ZBTB16- and vector-expressing cells were implanted in six-well plates at increasing concentrations (200,400, and 800 cells/well) and cultured for 14 days. Surviving colonies (≥ 50 cells/colony) were initially scanned with the CanoScan 8800F MOEL-85 scanner and counted after fixation with 4% paraformaldehyde, followed by staining with Gentian violet. Three independent replicates of each assay were performed.

### The soft agar colony formation assay

The soft agar assay was used to confirm cellular anchorage-independent growth as previously described [34]. Briefly, cells were cultured in 6-well plates at 37 °C with 0.35% top agarose containing 1 × 10^3^ cells and 1.2% bottom agarose harboring corresponding medium (RPMI 1640), supplemented with 10% fetal bovine serum (FBS) in all agarose solutions. Cell colonies were photographed and counted after 3 weeks of incubation. Each experiment was repeated three times.

### Flow cytometry (FCM)

Cell cycle and apoptosis were determined via flow cytometry (FCM) [44, 45]. To evaluate the cell cycle, cells were transfected, fixed, and stained by propidium iodide (PI). To analyze apoptosis, double staining was performed with annexin V-fluorescein isothiocyanate and PI. A CellQuest kit (BD Biosciences, CA, USA) was employed to assess FCM results. All experiments were performed in triplicate.

### Wound healing and Transwell® assays for cell migration and invasion

Wound healing and Transwell® assays were used to assess cell migration and invasion capacities as described previously [34, 46]. Assays were conducted using Transwell chambers (Corning Incorporated, 2 Alfred Road, Kennebunk ME 04043 USA) with 6.5 mm diameter inserts and a pore size of 8 μm. The Transwell membrane was coated with Matrigel glue (BD Biosciences) for detection of cell invasion. Images of cells on the lower surface of the chamber were obtained under a phase-contrast microscope (Leica) at 24 h after fixation and staining, followed by cell counting. All experiments were performed in triplicate.

### Tumor xenograft model in nude mice

The Animal Center of Chongqing Medical University certified all procedures involving mice and experimental protocols. Six BALB/c-nude mice aged 4–6 weeks were obtained from Beijing Huafu Biotechnology Co. Ltd. Immunocompromised female nude mice were used for xenograft studies. Stable MDA-MB-231 cells with or without ZBTB16 was digested to generate a single cell suspension. Each mouse was subcutaneously injected with 200 μl PBS containing 2.5 × 10^6^ suspended cells and primary tumor size measured every 2 days after 7 days of injection. The housing facility was maintained in keeping with national standards (Laboratory Animal-Requirements of Environment and Housing Facilities; GB14925-2010). Care of laboratory animals and experimental operations conformed to the Chongqing Management Approach of Laboratory Animals (Chongqing government order No.195). Tumor volume (mm^3^) was calculated as follows: volume = length × width^2^ × 0.52.

### Co-immunoprecipitation (Co-IP) and Western blot

Western blot analysis was performed as described previously [34, 47]. Aliquots of 40 μg protein lysates were separated via sodium dodecyl sulfate polyacrylamide gel electrophoresis (SDS-PAGE) and transferred onto polyvinylidene difluoride (PVDF) membranes (Bio-Rad, Hercules, CA, USA). Next, membranes were incubated with primary antibodies specific for ZBTB16 (#ab104854, Abcam), E-Cadherin (#1702-1; Epitomics), N-Cadherin (WL01047, Wanleibio, China), Vimentin (#2707-1; Epitomics), ZBTB28 (ab180084; Abcam), and BCL6 (sc-7388; Santa Cruz, Germany). Protein bands were examined using an Immobilon Western Chemiluminescent HRP Substrate kit (Millipore Corporation, Billerica, MA, USA).

The Co-IP assay was selected to analyze interactions of ZBTB16 with ZBTB28 and BCL6 proteins. BT549 cells stably expressing ZBTB16 were used for experiments. In brief, BT549 cells were transfected with ZBTB16 plasmid and total protein was extracted with ice-cold low salt lysis buffer, followed by incubation with anti-Flag M2 (#F3165,Sigma-ALDRICH), ZBTB28 (ab180084; Abcam), BCL6 (sc-7388; Santa Cruz, Germany), or IgG (#2729, Cell Signaling Technology) antibodies overnight on the Ferris wheel at a speed of 60 yards at 4 °C. The Millipore SigmaTm Pure ProteomeTm Protein A/G Mix Magnetic Bead System (#LSKAGAG10, Fisher Scientific, USA) was used to purify the Co-IP complex and beads were washed with low-salt lysis buffer. Western blot was applied to analyze the co-immunoprecipitation complex.

### Luciferase reporter assay

pGL3-BCL6 and pGL3-ZBTB28 wild-type/mutant plasmids were used for the reporter assays, as described previously [34]. Renilla plasmid was used as a control. After 48 h of transfection, lysis buffer (100 μl/well) was added and 20 μl sample detected using the Dual-luciferase reporter assay kit (Promega, Madison, WI), followed by 100 μl substrate (50 μl start + 50 μl stop). Light emission was quantified on the Infinite M200 PRO luminometer (Tecan, Austria) following the manufacturer’s manual.

### Immunohistochemistry (IHC) staining

Immunohistochemistry (IHC) was used to detect expression of ZBTB16 in paraffin-embedded primary breast tumors and paired paracancerous tissues as well as xenograft tumor tissues in mice. Tissues were sliced into 4-μm-thick sections and baked overnight at 65 °C, washed with PBS and stained with an Immunohistochemistry Kit (ZSGB-BIO, Beijing, China) according to the manufacturer’s instructions. Slides were incubated at 4 °C overnight (16-20 h) with Ki67 (#16667, Abcam) and ZBTB16 (#ab104854, Abcam) antibodies. The next day, slides were stained with DAB substrate (K176810E, ZSGB-BIO, China) for 30 s and counterstained with hematoxylin for 3 s. Images were examined under a microscope.

### Statistical analyses

All statistical analyses were performed with SPSS software (version 16, SPSS, Chicago, IL, USA). Two-tailed Student’s *t* tests, Mann-Whitney *U* test, and chi-squared test were conducted to determine *p* values. Data were considered significant at *p* < 0.05.

## Supplementary information

**Additional file 1: Figure S1.** (A). ZBTB16 expression status in tissues of normal adults. RNA integrity has been confirmed by GAPDH test shown in our previous publications. (B). ZBTB16, ZBTB28 and BCL6 expression in breast cancer cells, data from TCGA cancer dataset, accessed through cBioPortal (www.cbioportal.org).

**Additional file 2: Figure S2.** The correlations between ZBTB16 and EMT markers from the GCE database.

## Data Availability

The datasets used and/or analyzed during the current study are available from the corresponding author on reasonable request.

## Abbreviations

ZBTB16: Zinc finger and BTB domain-containing 16
ZBTB28: Zinc finger and BTB domain-containing 28
BCL6: B cell lymphoma 6 protein
ZBTB27: Zinc finger and BTB domain-containing 27
EMT: Epithelial-mesenchymal transition
BRCA1: Breast cancer type 1 susceptibility protein
BRCA2: Breast cancer type 1 susceptibility protein
PLZF: Promyelocytic leukemia zinc finger protein
ZFP145: Zinc finger protein 145
APL: Acute promyelocytic leukemia
RARα: Retinoic acid receptor alpha gene
TNBC: Triple-negative breast cancer
RFS: Poorer relapse-free survival
DMFS: Distant metastasis-free survival
OS: Overall survival
Aza: 5-Aza-2′-deoxycytidine
ER: Oestrogen receptor alpha
PR: Progesterone receptor
HER2: Human epidermal growth factor receptor 2
IHC: Immunohistochemistry
CO-IP: Co-immunoprecipitation
TSG: Tumor suppressor gene
