# Supplementary figures and images for: BTB/POZ zinc finger protein ZBTB16 inhibits breast cancer proliferation and metastasis through upregulating ZBTB28 and antagonizing BCL6/ZBTB27
Source: Clin Epigenetics. 2020 Jun 9;12:82. doi: 10.1186/s13148-020-00867-9 (PMC7285556; doi:10.1186/s13148-020-00867-9)

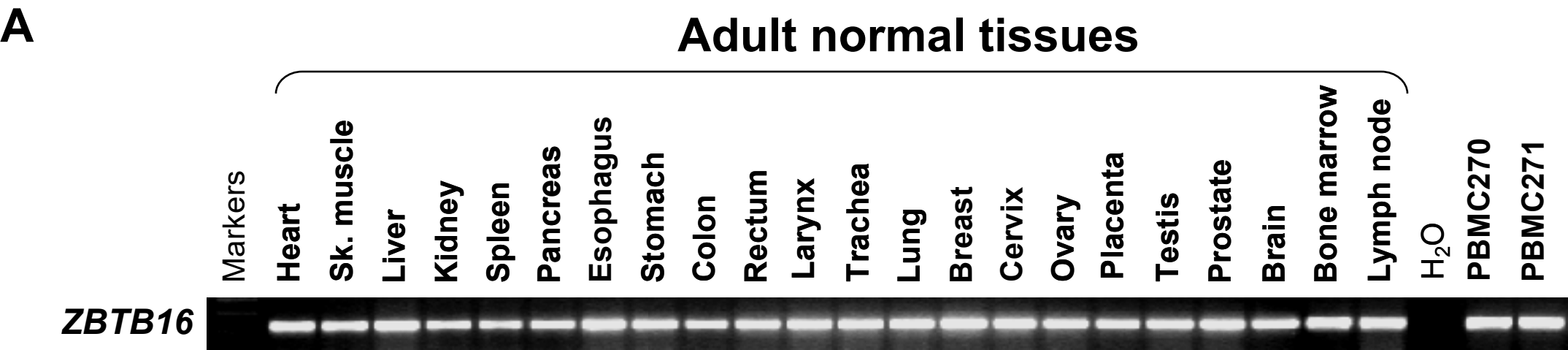

**B**

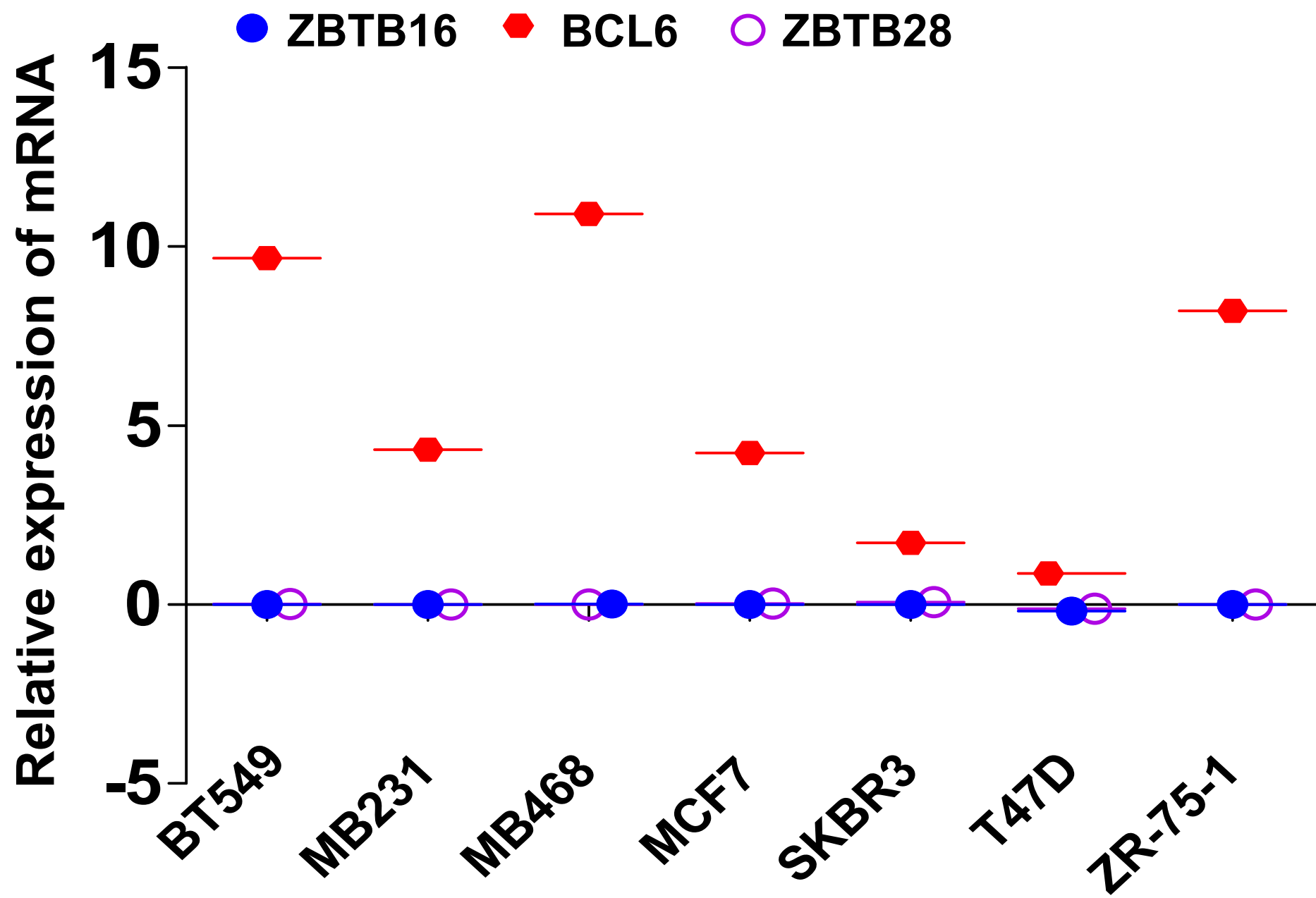

Supplement: Supplementary file 1 — Additional file 1: Figure S1. (A). ZBTB16 expression status in tissues of normal adults. RNA integrity has been confirmed by GAPDH test shown in our previous publications. (B). ZBTB16, ZBTB28 and BCL6 expression in breast cancer cells, data from TCGA cancer dataset, accessed through cBioPortal (www.cbioportal.org). [file 13148_2020_867_MOESM1_ESM.pdf]

(GSE36771, n=107)

**BrCa**

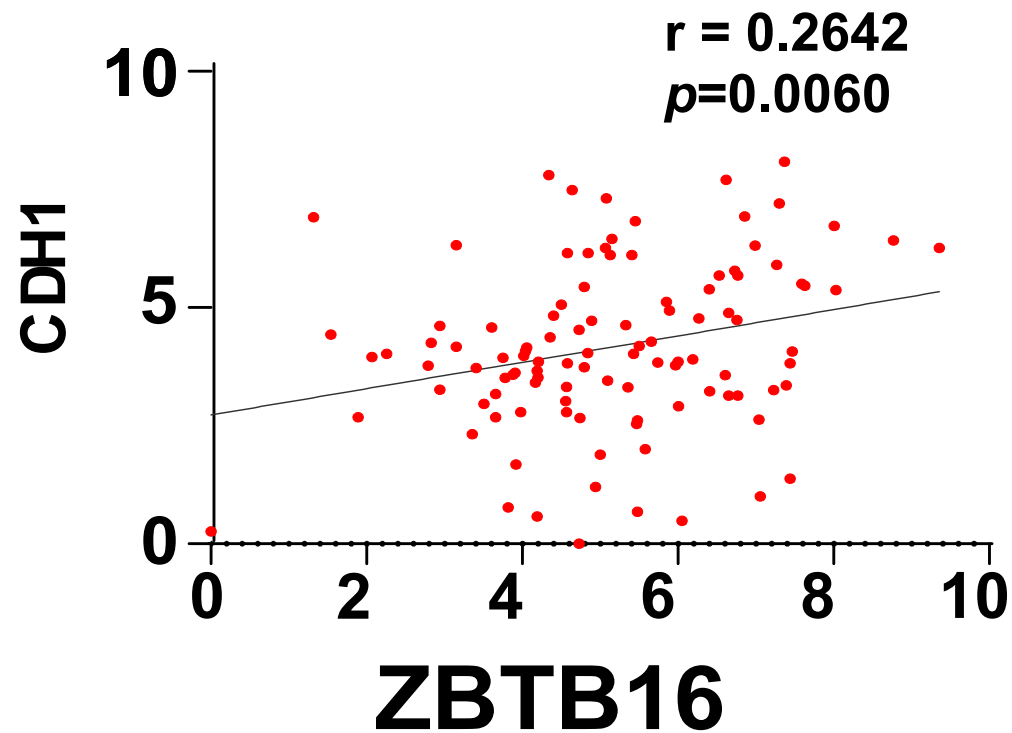

(GSE36771, n=107)

**BrCa**

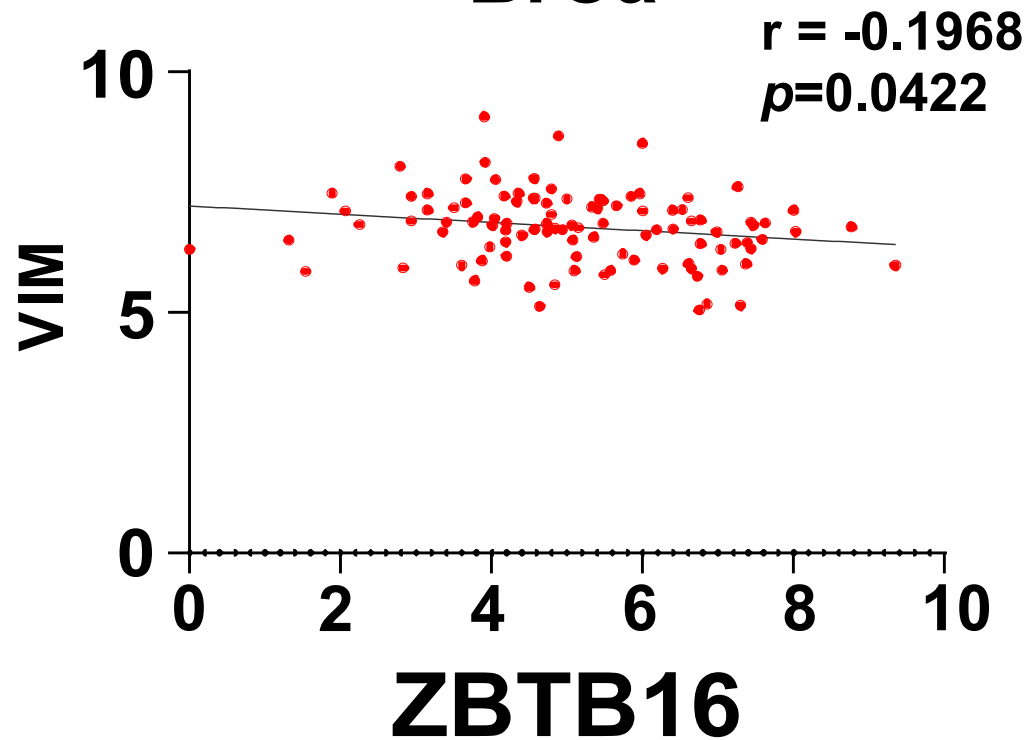

Supplement: Supplementary file 2 — Additional file 2: Figure S2. The correlations between ZBTB16 and EMT markers from the GCE database. [file 13148_2020_867_MOESM2_ESM.pdf]
